# Two non-homologous brain diseases-related genes, *SERPINI1 *and *PDCD10*, are tightly linked by an asymmetric bidirectional promoter in an evolutionarily conserved manner

**DOI:** 10.1186/1471-2199-8-2

**Published:** 2007-01-09

**Authors:** Ping-Yen Chen, Wun-Shaing W Chang, Ruey-Hwang Chou, Yiu-Kay Lai, Sheng-Chieh Lin, Chia-Yi Chi, Cheng-Wen Wu

**Affiliations:** 1 President's Laboratory and Institute of Cancer Research, National Health Research Institutes, Zhunan Town, Miaoli County 350, Taiwan, ROC; 2 Department of Life Sciences, National Tsing Hua University, Hsinchu City 300, Taiwan, ROC; 3 Department of Bioresources, Da-Yeh University, Changhua County 515, Taiwan, ROC

## Abstract

**Background:**

Despite of the fact that mammalian genomes are far more spacious than prokaryotic genomes, recent nucleotide sequencing data have revealed that many mammalian genes are arranged in a head-to-head orientation and separated by a small intergenic sequence. Extensive studies on some of these neighboring genes, in particular homologous gene pairs, have shown that these genes are often co-expressed in a symmetric manner and regulated by a shared promoter region. Here we report the identification of two non-homologous brain disease-related genes, with one coding for a serine protease inhibitor (*SERPINI1*) and the other for a programmed cell death-related gene (*PDCD10*), being tightly linked together by an asymmetric bidirectional promoter in an evolutionarily conserved fashion. This asymmetric bidirectional promoter, in cooperation with some cis-acting elements, is responsible for the co-regulation of the gene expression pattern as well as the tissue specificity of *SERPINI1 *and *PDCD10*.

**Results:**

While *SERPINI1 *is predominantly expressed in normal brain and down-regulated in brain tumors, *PDCD10 *is ubiquitously expressed in all normal tissues but its gene transcription becomes aberrant in different types of cancers. By measuring the luciferase activity in various cell lysates, their 851-bp intergenic sequence was shown to be capable of driving the reporter gene expression in either direction. A 175-bp fragment from nt 1 to 175 in the vicinity of *PDCD10 *was further determined to function as a minimal bidirectional promoter. A critical regulatory fragment, from nt 176-473 outside the minimal promoter in the intergenic region, was identified to contain a strong repressive element for *SERPINI1 *and an enhancer for *PDCD10*. These cis-acting elements may exist to help coordinate the expression and regulation of the two flanking genes.

**Conclusion:**

For all non-homologous genes that have been described to be closely adjacent in the mammalian genomes, the intergenic region of the head-to-head *PDCD10*-*SERPINI1 *gene pair provides an interesting and informative example of a complex regulatory system that governs the expression of both genes not only through an asymmetric bidirectional promoter, but also through fine-tuned regulations with some cis-acting elements.

## Background

In the human genome, there are over 550 genes coding for proteases with another 150 being protease inhibitors [1]. While the majority of protease genes belong to three different classes including serine, metallo- and cysteine proteases, a large portion (~25%) of human protease inhibitor genes belong solely to just one family: the serpin family.

The serpin family comprises hundreds of structurally homologous proteins that are present in both eukaryotic and prokaryotic organisms [2-4]. Its members include inhibitors of serine proteases as well as non-inhibitory members with other biological functions [5]. In humans, a total of 35 serpin-coding genes were identified and grouped into 9 subfamilies designated *SERPINA*, *SERPINB*, *SERPINC *and so on till *SERPINI *subfamily [2,3]. Each subfamily has its own specific chromosomal localization and well-conserved exon-intron arrangement. For example, nearly all members of the *SERPINA *subfamily are located at chromosome 14q32 and genes coding for the *SERPINB *subfamily are clustered at two distinct chromosomal loci: 6p25 and 18q21 [6-9]. Even for the last *SERPINI *subfamily, although consists of only two genes with one coding for a brain-associated protease inhibitor *SERPINI1 *[10] and another for a pancreas-specific protease inhibitor *SERPINI2 *[11], its two members are closely adjacent at chromosome 3q26 and share a perfectly conserved gene organization pattern [12].

*SERPINI1*, also known as neuroserpin or protease inhibitor 12, is an axonally secreted serine protease inhibitor found in the nervous systems [10]. It functions as a selective inhibitor of tissue-type plasminogen activator and is involved in a wide range of neuronal processes including synaptic plasticity [10,13], neuronal migration [14,15] and axogenesis [15,16]. Genetic variations of *SERPINI1 *have been reported to render the enzyme conformation locked as an inactive folding intermediate [17-20]. These mutant proteins then undergo spontaneous polymerization within neurons and cause a severe progressive neurodegenerative encephalopathy named FENIB (familial encephalopathy with neuroserpin inclusion bodies) [21,22]. In addition to the mutations at the DNA level, the mRNA expression of *SERPINI1 *was found to be down-regulated or lost in brain tumors [12]. These abnormalities, along with our latest findings that its adjacent gene *SERPINI2 *is also down-regulated in cancer [12], prompted us to study the regulatory mechanism of *SERPINI1 *gene in both normal and cancerous brain tissues. Interestingly, when the human genome sequence became available, it came to our attention that a gene termed *PDCD10 *was mapped to lie at less than 1 kb upstream of *SERPINI1 *in a divergent, head-to-head orientation.

*PDCD10 *was originally identified as *TFAR15 *(TF-1 cell apoptosis related gene-15) for being up-regulated after the induction of apoptosis by serum withdrawal in TF-1 human premyeloid cells [23]. It was later grouped into a collection of genes thought to be involved in many apoptotic responses and thus renamed as *PDCD10 *(programmed cell death 10 gene). *PDCD10 *is highly conserved among different species, with over 99% amino-acid identity among human, dog, mouse and chicken. Although its physiological role remains unclear, a recent study showed that inhibition of the nematode *PDCD10 *ortholog led to embryonic lethality in 40% of the embryos and a dumpy phenotype in postembryonic viable embryos [24]. Furthermore, *PDCD10 *mutations were identified in families with cerebral cavernous malformation, indicating that this gene may play a role in the development of cerebral vascular morphogenesis [25].

It is known that in prokaryotic and lower eukaryotic genomes, genes are often organized in a proximal pattern to facilitate gene co-expression and functional coupling. Increasing evidence suggests that many genes in mammalian genomes also tend to be in close physical vicinity. Recently, by conducting a genome-wide analysis of the human genome, Trinklein and his colleagues identified a class of head-to-head gene pairs whose transcriptional start sites are separated by less than 1 kb [26]. Since mammalian genomes are far more spacious than prokaryotic genomes, it seems unnecessary for two neighboring genes to be arranged in an adjacent head-to-head pattern unless they share a common regulatory system such as an intergenic bidirectional promoter. It is conceivable that bidirectional promoters of gene pairs may be able to co-regulate the genes in a more efficient manner. For example, for homologous gene pairs such as the pair of collagen type IV α1 and α2 genes [27] and the pair of ATP-binding cassette molecule *ABCG5 *and *ABCG8 *genes [28,29], their bidirectional promoters were shown to co-regulate the two genes thus ensuring the co-expressivity of the gene products. For non-homologous genes like *SERPINI1 *and *PDCD10*, however, the need for co-regulation is not as obvious. In addition, compared to all the other genes that are adjacently located to any of known *SERPIN *or *PDCD *genes, only the pair of *SERPINI1 *and *PDCD10 *genes were arranged in a head-to-head orientation and separated by an exceptionally short intergenic region. This close chromosomal proximity of *SERPINI1 *and *PDCD10 *and their divergent configuration are widely preserved in many species, implying that through the evolution there appears to have a need for *SERPINI1 *and *PDCD10 *to be tightly linked together to maintain gene co-regulation or functional association.

In this study, we present evidence showing that while *SERPINI1 *is predominantly expressed in brain and down-regulated in brain tumors, *PDCD10 *is ubiquitously expressed in all normal tissues but its gene transcription becomes aberrant in different types of cancers. We have also identified a 175-bp regulatory fragment within the intergenic region of *SERPINI1 *and *PDCD10 *that functions as a bidirectional promoter. Moreover, a critical fragment, from nt 176-473 outside the minimal promoter in this intergenic region, was found to possess a strong repressive activity for *SERPINI1 *and enhancing activity for *PDCD10*. These cis-acting elements may exist to coordinate the expression and regulation of the two flanking genes. To the best of our knowledge, of all non-homologous genes that have been reported to be closely adjacent in the human genome, the intergenic region of the head-to-head *PDCD10*-*SERPINI1 *pair at 3q26 exhibits by far the most complex regulatory function that governs the expression of both genes not only through an asymmetric bidirectional promoter, but also through regulations with some other cis-acting elements.

## Results

### Expression patterns of *SERPINI1 *and *PDCD10 *in normal and cancerous tissues

**Figure 1:**
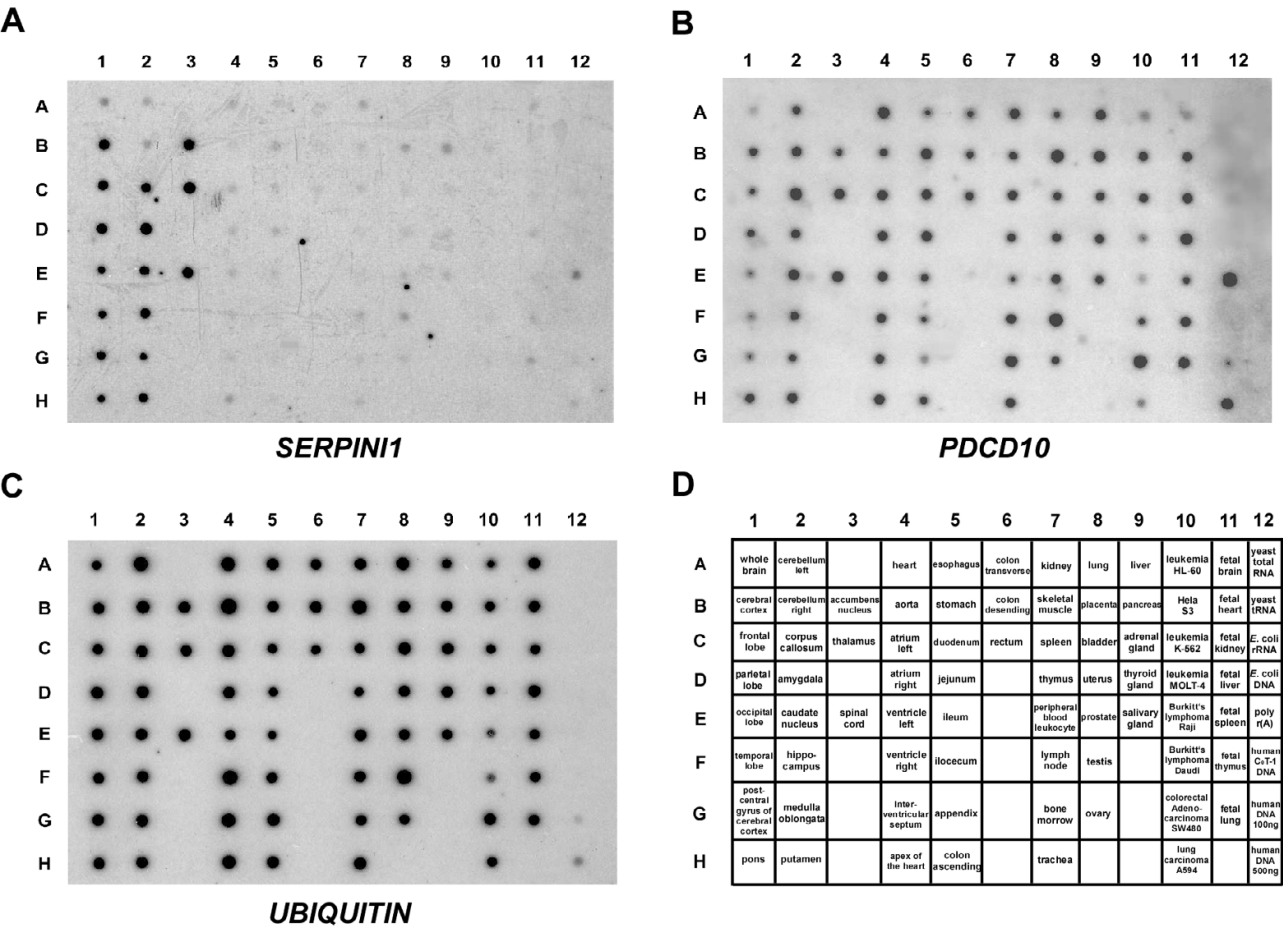
**Northern blot analysis of *SERPINI1 *and *PDCD10 *in various tissues**. Human MTE multiple tissue expression array II (BD Clontech) was hybridized with ^32^P-labeled probe against A) *SERPINI1*, B) *PDCD10*, and C) control ubiquitin, respectively. Panel D shows the individual designations of all tissues examined.

**Figure 2:**
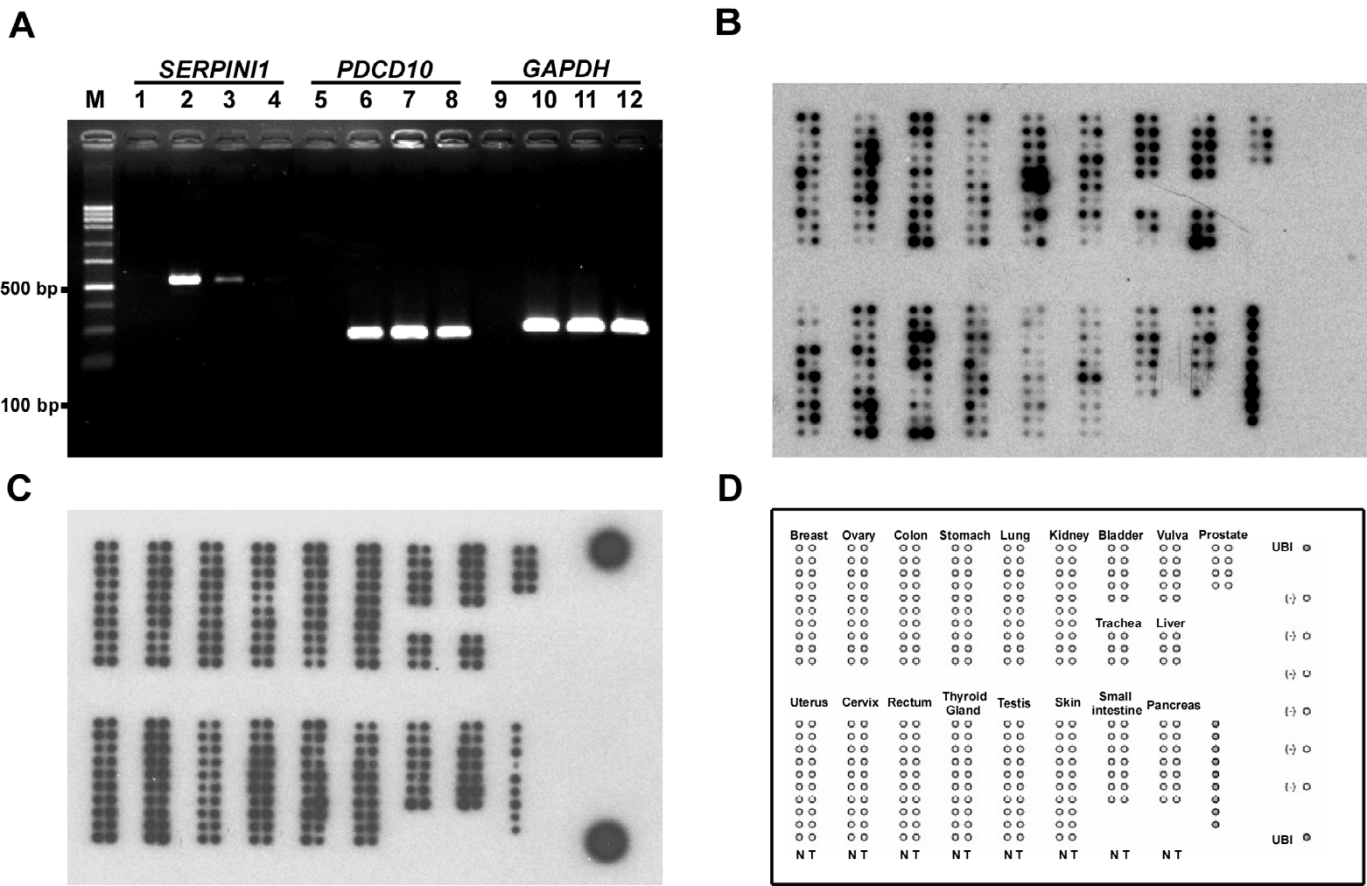
**Relative expression levels of *SERPINI1 *and *PDCD10 *between normal and tumorous tissues**. A) Semi-quantitative RT-PCR analysis of *SERPINI1 *and *PDCD10 *transcripts. RNAs used in this study were obtained from the normal human brain (Lanes 2, 6 & 10), human neuroglioma H4 cells (Lanes 3, 7 & 11) and human glioblastoma U-87 MG cells (Lanes 4, 8 & 12). M indicates the DNA size markers; and Lanes 1, 5 & 9 are the negative controls without templates in the PCR reactions. B & C) Northern hybridization analysis of *PDC*D10 (B) and ubiquitin (C) in paired normal/tumor tissue samples (BD Clontech). The array order of all tissues examined is shown in panel D.

In order to explore the distribution patterns of *SERPINI1 *and *PDCD10*, we performed Northern blot analysis on a human multiple tissue expression array of poly A+ RNAs. As illustrated in Fig. 1, while *SERPINI1 *expression was observed mainly in different parts of brain, hybridization of the same array with the *PDCD10 *probe revealed that it was ubiquitously expressed as a housekeeping gene in all tissues analyzed. Moreover, as *SERPINI1 *and *SERPINI2 *were both reported to be down-regulated during tumor development [12], it is rational to doubt that the expression of *PDCD10*, which is located in between the two *SERPINI *genes at chromosome 3q26, may also be altered in some tumors. Results from the semi-quantitative RT-PCR indicate that while *SERPINI1 *expression was negatively correlated with tumor progression, the mRNA level of *PDCD10 *was not much affected in brain tumor cells (Fig. 2). Furthermore, when we investigated the expression patterns of *PDCD10 *in other normal and cancerous tissues, we found that *PDCD10 *was up-regulated in various cancers including ovarian, stomach, lung, uterus and small intestine cancers (Fig. 2).

### Determination of the distance of the intergenic region between human *SERPINI1 *and *PDCD10 *genes

**Figure 3:**
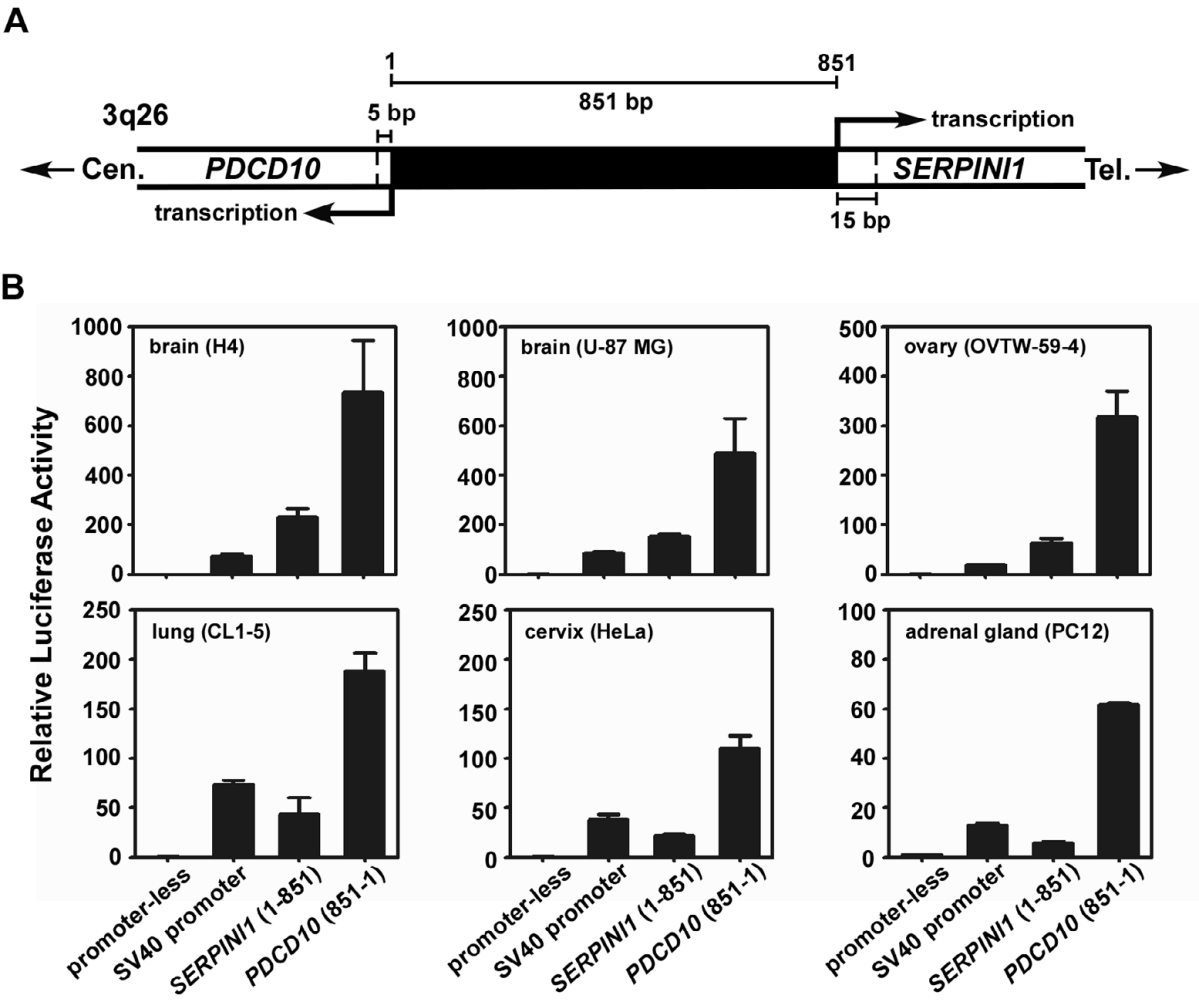
**Analysis of the intergenic distance between *PDCD10 *and *SERPINI1 *and its promoter activity in various cell lines**. A) The intergenic distance between *PDCD10 *and *SERPINI1 *was 851 bp, as determined from the results of 5'-RACE. The nucleotides between the two closest transcriptional start sites of *PDCD10 *and *SERPINI1 *are numbered from the centromeric (cen) end to the telomeric (tel) end. B) The promoter activity of the intergenic 851-bp fragment in either direction (toward *PDCD10 *or *SERPINI1*) was determined by the firefly luciferase assay in 6 different cell lines including human neuroglioma H4, human brain glioblastoma U-87 MG, human ovarian adenocarcinoma OVTW-59-4, human lung adenocarcinoma CL1-5, human cervical adenocarcinoma HeLa, and rat adrenal gland pheochromocytoma PC-12 cells.

**Figure 4:**
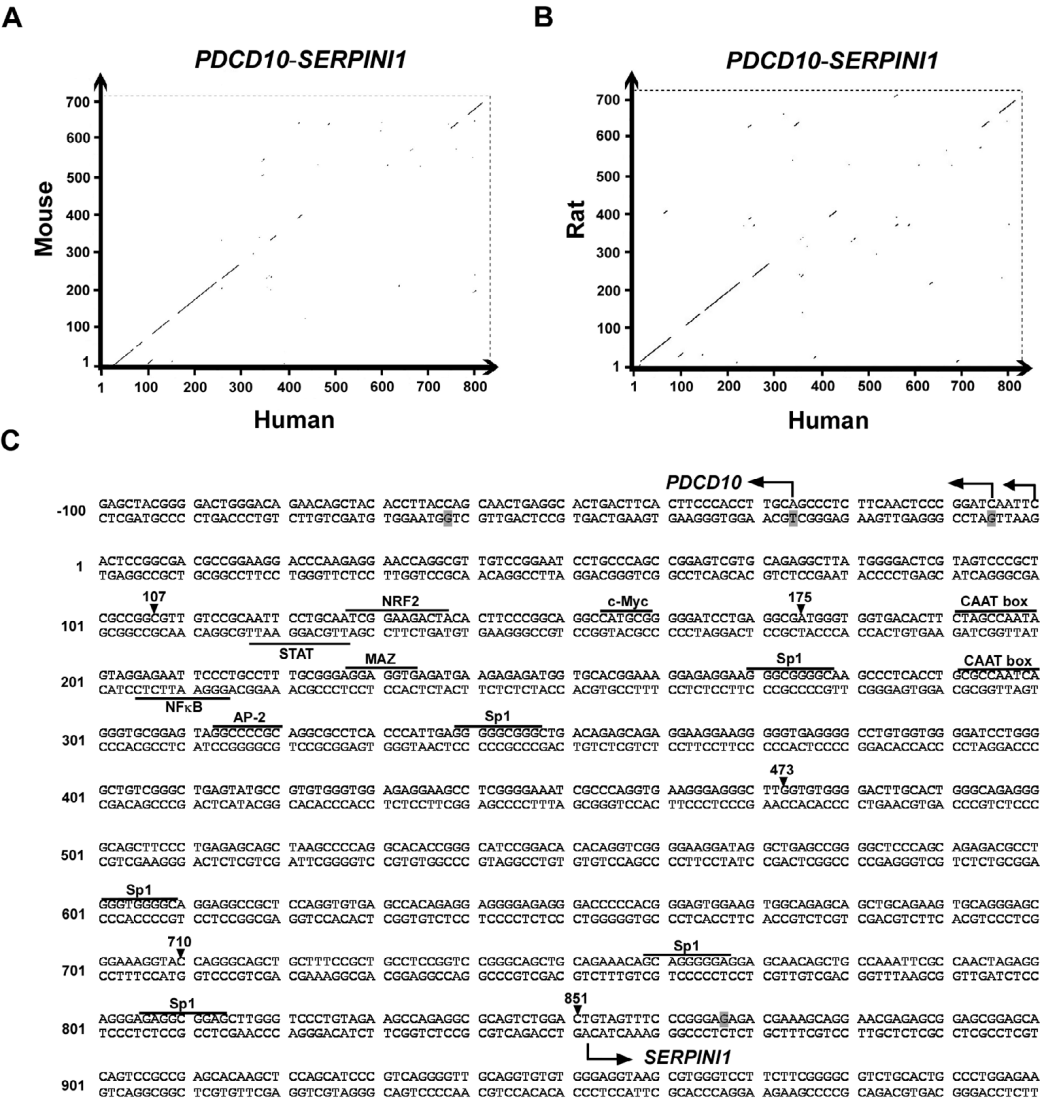
**Sequence analysis of the intergenic region of the divergent *PDCD10*-*SERPINI1 *gene pair**. Dot matrix alignment of the intergenic sequence of the human *PDCD10*-*SERPINI1 *pair with those of mouse (A) and rat (B). The units in both vertical and horizontal axis are the position numbers of the nucleotides in the intergenic region as shown in Fig. 3A. C) The complete nucleotide sequence of the intergenic spacer between human *PDCD10 *and *SERPINI1*. The putative transcription factor binding sites are underlined. The transcriptional start sites confirmed by 5'-RACE are indicated by bended arrows. The shaded nucleotides indicate the transcriptional start sites of the reference sequence reported by the NCBI database. The arrowheads indicate the cutting sites for the deletion assays described in Fig. 5.

After analyzing the distribution of mRNA transcripts for both *SERPINI1 *and *PDCD10 *genes, we then attempted to pinpoint their precise transcriptional start sites in order to determine the length of their intergenic spacer. As revealed by the human genome reference sequence, *SERPINI1 *[accession number EMBL:BC018043] and *PDCD10 *[accession number EMBL:AF022385] were mapped in a head-to-head configuration at 3q26 and separated by less than 1 kb. By performing 5'-RACE on human brain RNA, three transcriptional start sites for *PDCD10 *but only one transcriptional start site for *SERPINI1 *were identified. The closest transcriptional start site that we identified for *PDCD10 *and *SERPINI1 *were at 5 and 15 bp respectively upstream of the sites predicted by NCBI database, thus the shortest intergenic distance was recalculated to be 851 bp (Fig. 3 & 4).

### Characterization of the asymmetric bidirectional promoter activity of the intergenic region

Following the mapping of the transcriptional start sites, the 851-bp intergenic fragment constituting the putative regulatory region for both *SERPINI1 *and *PDCD10 *genes was analyzed for its transcriptional activities by driving the firefly luciferase reporter gene. The human cell lines utilized for luciferase reporter assays include brain neuroglioma H4, brain glioblastoma U-87 MG, ovarian adenocarcinoma OVTW-59-4, lung adenocarcinoma CL1-5, and cervical adenocarcinoma HeLa cancer cell lines. The rat adrenal gland pheochromocytoma PC-12 cell line was also used for comparison as it had previously been chosen for the study of mouse *Serpini1 *promoter [30]. The full-length 851-bp fragments in either direction toward *SERPINI1 *or *PDCD10 *were cloned into the reporter vector (pGL3-Basic). By measuring the luciferase activity in various cell lysates after transient transfection, the 851-bp intergenic sequence was shown to be capable of driving the reporter gene expression in either direction (Fig. 3). The promoter activities in the direction of *PDCD10 *in all cells tested were approximately 4 to 10-fold higher than those of *SERPINI1*. Moreover, the promoter activities toward the *SERPINI1 *direction were higher in brain tumor H4 and U-87 MG cells than those in other test cells, and they were nearly 3-fold higher than the positive control, the viral SV40 minimal promoter (Fig. 3). These results indicate that the 851-bp intergenic region is able to function as a bidirectional promoter to control *SERPINI1 *and *PDCD10 *expressions, and some regulatory elements within this region may asymmetrically regulate the transcription of both genes.

### Interspecies comparison of the 851-bp intergenic region and analysis of the putative cis-regulatory elements within this region

To understand whether the head-to-head configuration of *SERPINI1 *and *PDCD10 *is a unique feature only in human or if it is evolutionary conserved in other mammals, we aligned the homologous regions between these two genes among human, mouse and rat by the Vector NTI program (InforMax, Bethesda, MD, USA). The results revealed that both the gene order and the compact head-to-head arrangement are conserved in different species (data not shown), and that within the intergenic region there is an approximately 400-bp fragment near the vicinity of *PDCD10 *with the highest percent identity (Fig. 4A). Based on the nucleotide sequence shown in Fig. 4B, the 851-bp intergenic region has a GC content of 65%, which is much higher than the average of the entire human genome (41%). While no TATA-box is observed in this region, two CAAT boxes (consensus GG^T^/_C_CAATCT) are noted in the most conserved 400-bp fragment with a 100-bp distance from each other. In addition, within this highly conserved fragment, there are one neuron-specific AP-2, one c-Myc and one NFκB binding motives as well as several binding sites for the general transcription factor Sp1 as predicted by MatInspector (Genomatix; ) and TFSEARCH programs (Computational Biology Research Center; ). The presence and conservation of these putative binding motives suggest that some of these transcription factors must possess an important evolutionary significance and some yet-unknown regulatory demands.

### Identification of the key regulatory region of the bidirectional promoter by deletion analysis

**Figure 5:**
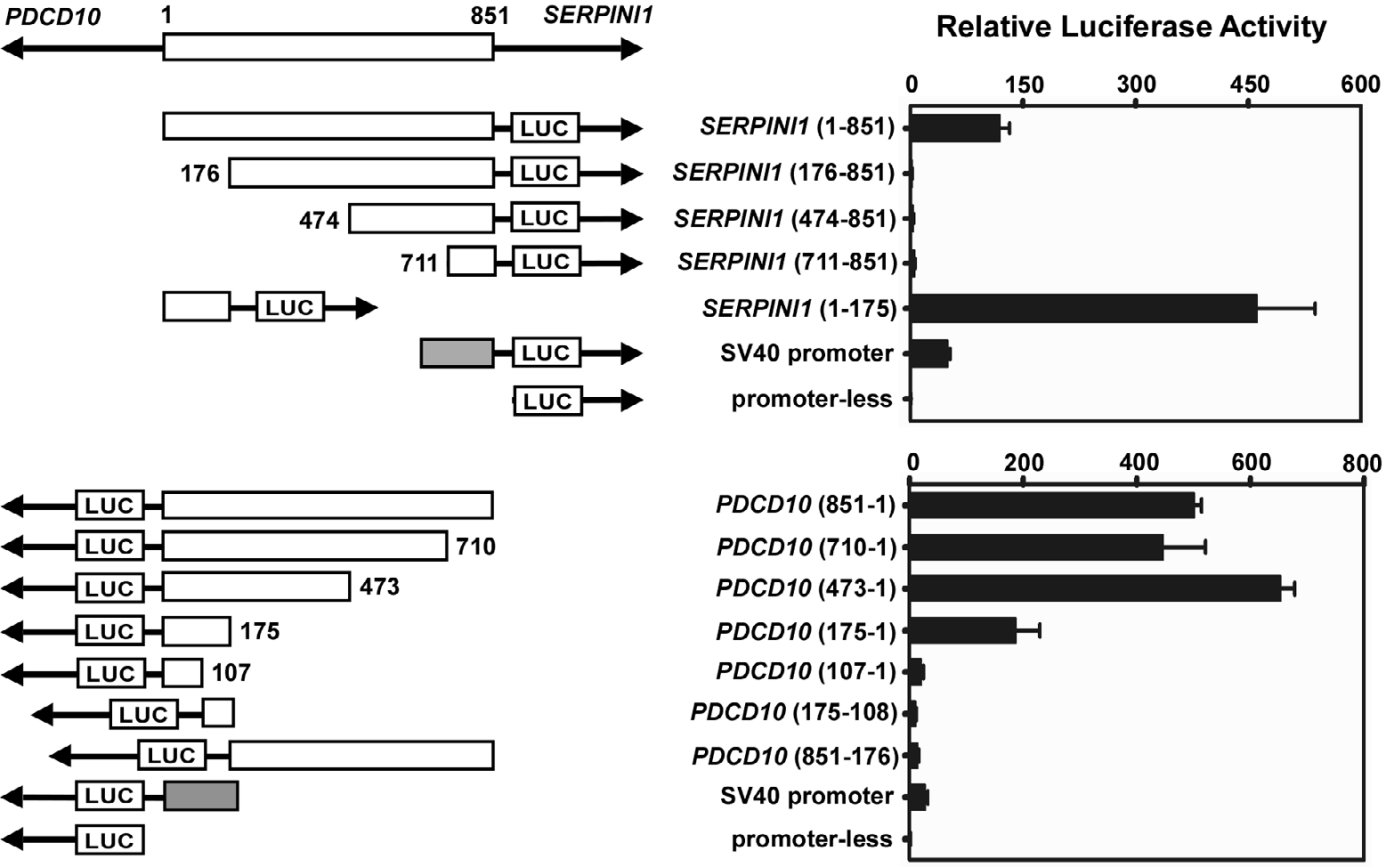
**Identification of the minimal bidirectional promoter region of the *PDCD10*-*SERPINI1 *gene pair**. The 5'- and 3'-deleted putative promoter fragments were individually cloned into the upstream of the firefly luciferase gene (LUC) in the pGL3-Basic vector followed by transient transfection into human neuroglioma H4 cells. The pRL-TK plasmid carrying *Renilla *luciferase gene was co-transfected as the internal control. The relative firefly luciferase activities were normalized to the *Renilla *luciferase activities to correct the transfection efficiency. The length and the position of each promoter insert are shown in scale. The numbers denote the positions of nucleotides with respect to +1, the first intergenic nucleotide next to the transcriptional start site of *PDCD10*, and +851, the intergenic nucleotide right next to the transcriptional start site of *SERPINI1*. The empty boxes represent inserted intergenic sequences. The shaded boxes represent the SV40 promoter.

To identify the key regulatory DNA sequences responsible for *SERPINI1 *and *PDCD10 *gene expressions, we constructed a series of plasmids containing various intergenic promoter regions derived from the 851-bp sequence. By using the same reporter system addressed in Fig. 3, the luciferase activities of these constructs were assessed by transient transfection into H4 cells. Due to the neuron-specific expression of the *SERPINI1 *gene, the promoter activity of *SERPINI1 *in neuron cells is higher than those in cells from other tissues. Thus, H4 cells were used as a vehicle for studying the regulatory mechanism of *SERPINI1 *promoter. As shown in Fig. 5, removal of the sequence from nt 1 to 175 completely abolished luciferase activity compared to the full-length 851-bp fragment in the *SERPINI1 *direction, suggesting that this 175-bp fragment is important for the control of *SERPINI1 *gene expression. This fragment alone was also capable of driving the reporter gene expression in the *PDCD10 *direction. Further splitting of this bidirectional promoter region into two parts, i.e. the sequence from nt 1 to 107 or from nt 108 to 175, resulted in a dramatic loss of luciferase activity. Surprisingly, deletion of the fragment of nt 176 to 851 increased more than 3-fold of the luciferase activity relative to that of the 851-bp fragment in the *SERPINI1 *direction but only kept a ~40% remaining of the luciferase activity in the *PDCD10 *direction (Fig. 5). These results strongly indicate that within the fragment of nt 176-851 there likely exist a repressive element that can regulate *SERPINI1 *expression and an enhancer that can modulate *PDCD10 *expression. Based on these findings, we conclude that a 175-bp minimal promoter region from nt 1 to 175 near the vicinity of *PDCD10 *and a regulatory region outside the 175-bp fragment from nt 176 to 851 may coordinately regulate the gene transcriptions of *SERPINI1 *and *PDCD10*.

### Possible locations of the repressive element for *SERPINI1 *and the enhancer for *PDCD10*

**Figure 6:**
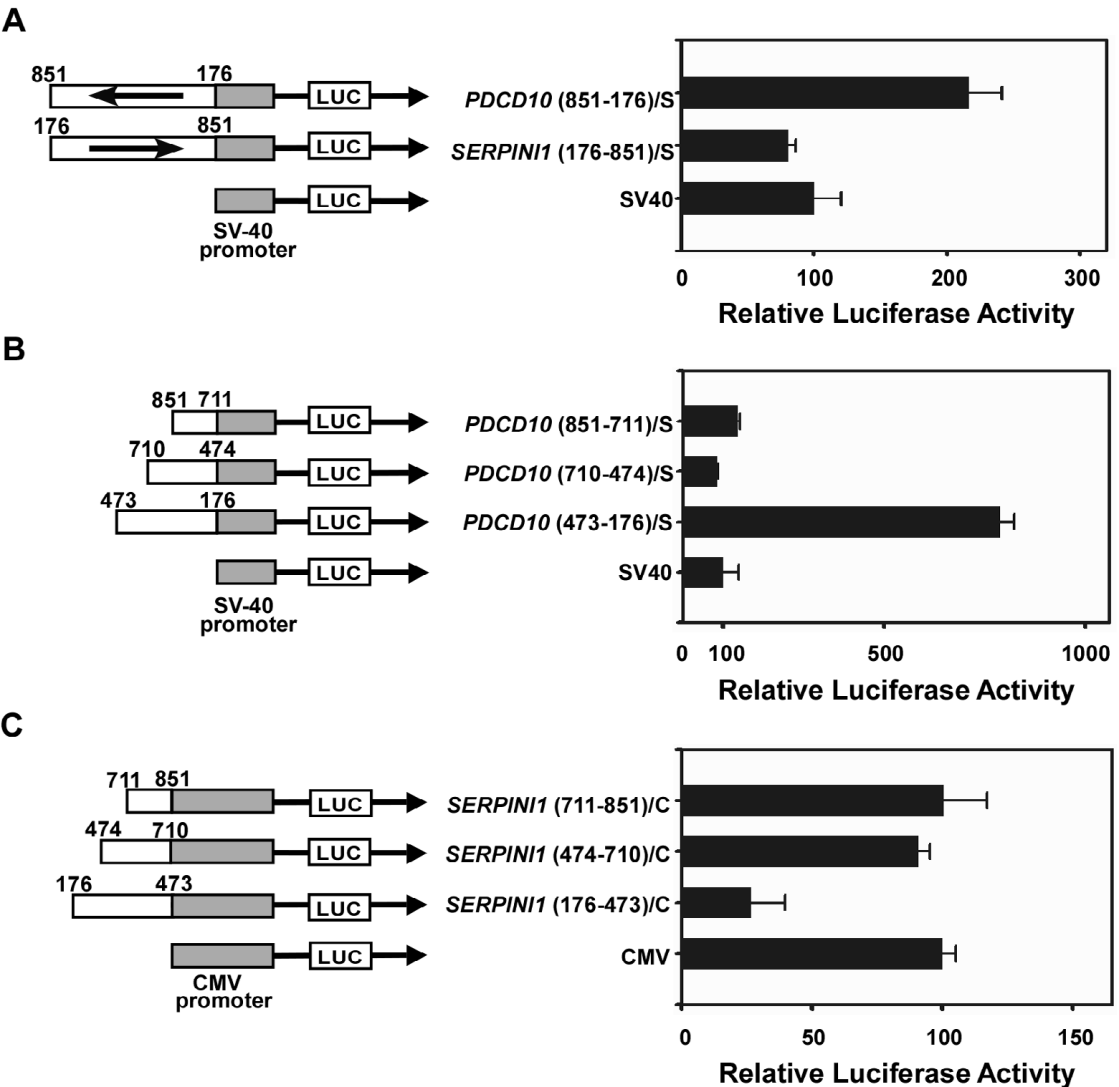
**Identification of the enhancer and the repressive element within the intergenic region of the *PDCD10*-*SERPINI1 *gene pair**. Various intergenic fragments were cloned upstream of either the SV40 or CMV promoter and of a luciferase reporter gene in the pGL3 vector. All constructs were transiently transfected into human neuroglioma H4 cells and the luciferase activity was measured. The resulting promoter activity was calculated relative to the control SV40 or CMV promoter activity which is artificially set at 100. A) The potential regulatory sequences from nt 176 to 851 of the intergenic region were cloned upstream of the SV40 promoter and their effects on the activity of the SV40 promoter were examined. B) Three intergenic fragments (nt 851-711, nt 710-474 and nt 473-176) in the *PDCD10 *direction were individually cloned upstream of the SV40 promoter to assess their effects on the activity of the SV40 promoter. C) Three different intergenic fragments (nt 711-851, nt 474-710 and nt 176-473) in the *SERPINI1 *direction were individually cloned upstream of the CMV promoter in a modified pGL3-Promoter vector. Their effects on the activity of the CMV promoter were then investigated. The numbering system for nucleotide position is the same as those described in Fig. 5. The length and the position of each promoter insert are shown in scale. In the names of constructs, S denotes the SV40 promoter and C denotes the CMV promoter.

From the experiment described in Fig. 5, there seems to exist a regulatory fragment from nt 176 to 851 which possesses no promoter activity but appears to contain a repressive element for *SERPINI1 *and an enhancer for *PDCD10*. Thus, we cloned this fragment in two opposite directions into the upstream of the SV40 promoter in a pGL3-Promoter vector, and examined their effects on the SV40 promoter. As expected, the fragment of nt 176-851 in the *PDCD10 *direction was able to increase the SV40 promoter activity for approximately 2.2-fold (Fig. 6A). This observation confirmed the existence of an enhancer for *PDCD10 *as seen in Fig. 5. However, there was no increase of the SV40 promoter activity in the direction of *SERPINI1*. This phenomenon was possibly a compromising effect resulted from the coordination between the enhancer and the repressive element within the regulatory region; the extent of such effect may also vary for using different heterologous promoters.

To further identify the enhancer responsible for *PDCD10*, three intergenic fragments including 1) the nt 176-473 fragment, 2) the nt 474-710 fragment, and 3) the nt 711-851 fragment toward the *PDCD10 *direction were individually cloned into the upstream of the SV40 promoter and assayed for their promoter activity in H4 cells. The results showed that only the nt 473-176 fragment was able to increase the SV40 promoter activity by 6 to 10-fold (Fig. 6B). The same fragment toward the *SERPINI1 *direction had no such effect on the SV40 promoter activity (data not shown). Hence, there is an orientation-dependent enhancer located in the fragment of nt 176-473 and acting on the transcription of *PDCD10*. As for the search of the repressive element for *SERPINI1*, the same fragments but in opposite direction were individually cloned into a modified pGL3-Promoter vector containing a CMV promoter, a stronger promoter than the SV40 and better for repressive activity studies. The assay results demonstrated that only the fragment from nt 176-473 toward the *SERPINI1 *direction possessed a repressive ability which inhibited ~70% of the CMV promoter activity (Fig. 6C). Nonetheless, the same fragment but in the opposite orientation (toward the *PDCD10 *direction) could only enhance the CMV promoter activity for approximately 2-fold (data not shown). These findings signify that a critical fragment from nt 176-473 outside the minimal promoter possesses a strong repressive activity for *SERPINI1 *and enhancing activity for *PDCD10*.

## Discussion

It has long been suggested that the human serpin genes are evolved from the same ancestor through intra- and interchromosomal duplications to develop into different clusters [31-33]. Interestingly, when the human genome sequence was unveiled a few years ago, several non-homologous genes were found to be mapped into the serpin gene clusters. One of these genes, *PDCD10*, not only forms a head-to-head organization with *SERPINI1 *at chromosome 3q26 but also shares a tiny promoter with it. The organization of this divergent gene pair is well preserved in many species throughout the evolution, indicating that *PDCD10 *and *SERPINI1 *need to be tightly together to maintain the gene co-regulation or their functional association. Further sequence analysis on the intergenic region revealed a 400-bp fragment near the vicinity of *PDCD10 *is highly similar among human, mouse and rat, a result strongly indicating that the transcriptional regulation mechanisms of these two genes are evolutionary conserved among different species. Indeed, by performing a series of luciferase assays, a 175-bp fragment from nt 1 to 175 in the vicinity of *PDCD10 *was determined to function as a minimal promoter of both genes (Fig. 5). The same 175-bp minimal promoter was also observed (data not shown) when we utilized the human intergenic fragment to perform the promoter analysis in rat adrenal gland PC12 cells [30]. In addition, we detected the existence of an important regulatory fragment from nt 176 to 473 in the vicinity of *PDCD10 *that possesses a repressive activity for *SERPINI1 *(Fig. 6). These findings are different from a previous study on mouse *Serpini1 *[30] claiming that a 200-bp segment near the transcription initiation site of *Serpini1 *was responsible for promoter activity. There are at least two possible reasons for the discrepancy observed in human and mouse *SERPINI1 *promoter. First, the constructs used for promoter analysis of mouse *Serpini1 *were all derived from 5' deletions of the full-length promoter region. These constructs might have contained the repressive element and/or lost the minimal promoter, and thus have very low promoter activities which could not be differentiated from each other. Secondly, different cell lines chosen might result in different conclusions; particularly the rat adrenal gland cell line PC12 has very low expression of *Serpini1*.

Trinklein and colleagues have pointed out that divergently transcribed gene pairs whose transcriptional start sites are separated by less than 1 kb represent more than 10% of genes in the whole human genome [26]. Such a high proportion indicates that there is a biological importance for those adjacent gene pairs even through a long evolution. Two feasible explanations for the existence of bidirectional promoters in the mammalian genome were recently given by Zhang and colleagues [34]. The first explanation is that they are the ancestral sequences survived in evolution. A bidirectional promoter, like the one for *PDCD10 *and *SERPINI1*, with the characteristics of TATA-less, GC-rich and multiple Sp1 sites was thought to be the preserved ancestral sequences. The second explanation is that the genes sharing the bidirectional promoter are functionally related. From the sequence alignment data, we observed that this highly conserved promoter sequence is maintained throughout the evolution. Besides, there are evidences indicating that *PDCD10 *and *SERPINI1 *are both related to brain diseases. Therefore, we suggest that the compact adjacent arrangement for *PDCD10 *and *SERPINI1 *might be due to the possibility that they are involved in the same biological pathway.

Apart from the *PDCD10*-*SERPINI1 *gene pair at chromosome 3q26, there was another human head-to-head gene pair found to be associated with brain diseases [35]. This gene pair, consisting of the parkinsonism-related parkin gene (*PARK2*) and parkin co-regulated gene (*PACRG*) at chromosome 6q25.2-27, has many features in common with the *PDCD10*-*SERPINI1 *pair. Like *SERPINI1 *and *PDCD10*, *PARK2 *and *PACRG *are also non-homologous. *PARK2*, like *SERPINI1*, has its tissue specificity and is mainly expressed in brain, heart, skeletal muscle and kidney. *PACRG *is expressed in all tissues like *PDCD10*. The bidirectional promoter of *PACRG*-*PARK2 *is also TATA-less, GC-rich, and with multiple Sp1 binding sites [35,36]. Furthermore, the genetic mutations of *SERPINI1 *and *PARK2 *are able to accelerate the formation of cellular inclusion bodies and to cause the dementia symptom [18,21,37]. Therefore, we suggest that brain, being the most sophisticated organ which controls almost all responses of the whole body, may use bidirectional promoters to achieve gene expressions more efficiently, especially in the pathogenesis of its diseases.

Gene expression and tissue specificity are often regulated by transcriptional activators or repressors [38-40]. In the case of the *PDCD10*-*SERPINI1 *gene pair, it is apparent that these two genes are not co-expressed genes (Fig. 1). This phenomenon can be best explained by the existence of a critical fragment, from nt 176 to 473 of the intergenic sequence, which contains an enhancer and a repressive element to coordinately regulate the transcriptions of both genes (Fig. 5 & 6). The intergenic enhancer is orientation-dependent exerting its effect on the promoter of *PDCD10 *in the native context (Fig. 5). It also works in an orientation-dependent manner with heterologous promoters (Fig. 6A & 6B). Previously, a similar orientation-dependent intergenic enhancer had been reported in a mouse gene pair: the divergent *Cryab *(αB-crystallin) and *Hspb2 *(heat shock 27 kDa protein 2) pair [41]. However, this is the first time that such an enhancer is identified in the human genome. Moreover, the neuron-specificity of *SERPINI1 *and its down-regulation in brain tumorigenesis may be accounted for by a repressive element located in the same critical regulatory fragment (Fig. 5 & 6). Together these results suggest that the fine coordination between the orientation-dependent cis-control elements may play an important role in the asymmetrical expressions of *PDCD10 *and *SERPINI1 *in different tissues. In addition to the cis-control elements, the possible involvement of certain trans-acting factors in this bidirectional promoter region need to be further elucidated.

## Conclusion

Here we present evidence that two non-homologous brain diseases-related genes, *SERPINI1 *and *PDCD10*, are tightly linked in a divergent configuration by a bidirectional promoter in an evolutionarily conserved fashion. Within their tiny intergenic region, a segment from nt 1 to 175 near the vicinity of *PDCD10 *was identified to function as a minimal promoter to efficiently drive the expressions of the two flanking genes. A critical regulatory fragment from nt 176 to 473 was further determined to contain a repressive element for *SERPINI1 *and an enhancer for *PDCD10 *that coordinately regulate the transcriptions of both genes. For all the non-homologous genes that have been described to be closely adjacent in the human genome, the intergenic region of the head-to-head *PDCD10*-*SERPINI1 *gene pair at 3q26 exhibits an interesting and informative example of a complex regulatory system that governs the expression of both genes not only through an asymmetric bidirectional promoter, but also through fine-tuned regulations with some cis-acting elements.

## Methods

### Cell culture

All culture media, except RPMI 1640 medium, were purchased from Gibco Invitrogen (Carlsbad, CA, USA) and supplemented with 10% fetal bovine serum (FBS) (HyClone, Logan, UT, USA). H4 (human neuroglioma) and OVTW-59-4 (human ovarian adenocarcinoma) cells were cultured in DMEM (Dulbecco's modified Eagle's medium) medium, whereas U-87 MG (human brain glioblastoma) and HeLa (human cervical adenocarcinoma) cells were grown in minimum essential medium (MEM). CL1-5 (high-metastatic human lung adenocarcinoma) cells were cultured in RPMI-1640 medium (Hyclone) supplemented with 0.5 mM L-glutamine and 10% FBS. PC-12 (rat adrenal gland pheochromocytoma) cells were cultured in 85% RPMI-1640 supplemented with 5% FBS and 10% horse serum. All cell lines were maintained at 37°C in a humidified atmosphere of 5% CO_2 _in air.

### Northern blotting

Northern hybridizations were carried out mainly as described previously [12]. In brief, the probe for *SERPINI1 *was amplified from the FirstChoice PCR-Ready human brain cDNA (Ambion, Austin, TX, USA) using the forward primer 5'-CTGGAAGTCGCAGTTTAGGCCTGAA-3' and the reverse primer 5'-TGGATGGTCGACAATAACTTGAGGA-3' to produce a 559-bp fragment under the following conditions: 1 cycle of 94°C for 5 min; 32 cycles of 94°C for 20 sec, 55°C for 20 sec, and 72°C for 1 min; and 1 cycle of 72°C for 10 min. The probe for *PDCD10 *was amplified from the same cDNA using the forward primer 5'-TCCATGGTTTCTATGCCCCT-3' and the reverse primer 5'-TTTGGTGTTCAAGTGCCCTG-3' to produce a 445-bp fragment under the following conditions: 1 cycle of 94°C for 5 min; 35 cycles of 94°C for 30 sec, 55°C for 30 sec, and 72°C for 30 sec; and 1 cycle of 72°C for 10 min. All PCRs were performed on a T3 thermocycler (Whatman Biometra, Goettingen, Germany) using Ex Taq polymerase (Takara, Otsu, Singa, Japan) and subjected to sequencing on an ABI PRISM 3700 automated sequencer (Applied Biosystems, Foster City, CA, USA) by the core facility of the National Health Research Institutes (Zhunan, Taiwan). The sequence-verified probes, including a cDNA probe for ubiquitin supplied by the manufacturer and used as a control, were labeled with [α-^32^P]dCTP using the Rediprime II DNA labeling system (Amersham/GE Healthcare, Piscataway, NJ, USA) followed by hybridization against either the human MTE multiple tissue expression array or cancer profiling array (BD Bioscience Clontech, Palo Alto, CA, USA) in an ExpressHyb hybridization solution (BD Bioscience Clontech) for 4 h at 65°C. The arrays were then washed and exposed to X-ray film for detection. Each Northern blot analysis was repeated at least three times to confirm reproducibility of the results.

### Semi-quantitative reverse transcription polymerase chain reaction (RT-PCR)

Total RNAs were extracted from two human brain tumor cell lines, H4 and U-87 MG, using TRIZOL reagent (Gibco Invitrogen) followed by DNase (Ambion) treatment to eliminate potential genomic DNA contamination. For control, total RNA of normal brain tissue was obtained from Clontech. Complementary DNA (cDNA) was synthesized from 5 μg of total RNA using oligo(dT) priming (Roche Molecular Biochemicals, Indianapolis, IN, USA) and MMLV reverse transcriptase (Epicentre Technologies, Madison, WI, USA). After a 1-h incubation at 37°C, the reaction mixtures were heat inactivated at 92°C for 10 min and then applied as templates to amplify cDNAs specific for *SERPINI1 *(amplified with the forward primer 5'-CTTGCAATGGGAATGATGGAACTTG-3' and the reverse primer 5'-CGAAATCATGTCCACTTGTGTTCAT-3' at 1 cycle of 94°C for 5 min; 35 cycles of 94°C for 30 sec, 55°C for 30 sec, and 72°C for 90 sec; and 1 cycle of 72°C for 10 min) and for *PDCD10 *(amplified with the forward primer 5'-TCCATGGTTTCTATGCCCCT-3' and the reverse primer 5'-TTTGGTGTTCAAGTGCCCTG-3' at 1 cycle of 94°C for 5 min; 35 cycles of 94°C for 30 sec, 55°C for 30 sec, and 72°C for 30 sec; and 1 cycle of 72°C for 10 min). *GAPDH *serving as internal control was amplified using the primer set 5'-CCTGCACCACCAACTGCTTA-3' and 5'-ACCCTGTTGCTGTAGCCAAA-3' to allow comparison of RNA levels among different samples. The PCR conditions for *GAPDH *were the same as those for *PDCD10*.

### 5'-Rapid amplification of cDNA ends (RACE)

The 5'-RACE analysis was performed using the FirstChoice human brain RNA ligase-mediated (RLM)-RACE kit (Ambion). Two RNA adaptor primers were employed as forward primers according to the manufacturer's instruction. The reverse primers for the 5'-RACE of *SERPINI1 *were 5'-TAGGCTGTCATATCCCATTGAGTGG-3' for the outer primer and 5'-CAAGTTCCATCATTCCCATTGCAAG-3' for the inner primer. The reverse primers for the 5'-RACE of *PDCD10 *were 5'-CTCAGCTTCATTCTTCATCTCTTC-3' for the outer primer and 5'-CCTCATTCAAAAGCCAACTACAG-3' for the inner primer. Both the first and second rounds of PCR were performed with 1 cycle at 94°C for 3 min; 35 cycles at 94°C for 30 sec, 60°C for 30 sec, and 72°C for 30 sec; and 1 cycle at 72°C for 7 min. The PCR products were cloned into a T-tailed vector pGEM-T-Easy (Promega, Madison, WI, USA) followed by sequencing to determine the 5'-ends of *SERPINI1 *and *PDCD10 *transcripts.

### Construction of the reporter gene plasmids

All restriction enzymes were purchased from Takara. Genomic DNA was purified from human whole blood samples using the QIAamp Blood kit (Qiagen, Hilden, Germany). The various lengths of the intergenic segment between *SERPINI1 *and *PDCD10 *were obtained by PCR using the primers listed in Table 1 under the following condition: 1 cycle of 94°C for 5 min; 35 cycles of 94°C for 30 sec, 55°C for 30 sec, and 72°C for 30 sec; and 1 cycle of 72°C for 10 min. The amplified DNA fragments were then cloned into one of the three vectors: pGL3-Basic, pGL3-Promoter, or pGL3-Promoter with the original SV40 promoter being replaced by a CMV promoter.

### Transient transfection and promoter assays

Cells were first seeded in 12-well culture plates at a density of 1.8 × 10^5 ^cells per well. Cells were then co-transfected with 3 μl of Lipofectamine 2000 (Gibco Invitrogen) and a mixture of 525 ng of DNA consisting of 500 ng of the tested pGL3 promoter constructs and 25 ng of the pRL-TK *Renilla *luciferase reporter plasmids (Promega) in a serum-free medium. After a 3-h incubation at 37°C, the transfection medium was replaced with fresh medium supplemented with 10% FBS and cells were incubated for another 24 h. After incubation, cells were washed twice with PBS and lysed by reporter lysis buffer (Promega). Cellular debris was removed after centrifugation at 10,000 × g for 5 min at 4°C. Cell extracts were then assayed for luciferase activity using a SIRIUS luminometer (Berthold, Bad Wildbad, Germany). In each experiment, cells were transfected with pGL3-Basic and pGL3-Promter plasmids that served as negative and positive control, respectively. To normalize transfection efficiency, the pRL-TK plasmids were co-transfected as an internal control.

## Abbreviations

The abbreviations used are: SERPINI1, serine protease inhibitor, clade I, member 1; PDCD10, programmed cell death 10; PARK2, parkin; PACRG, parkin co-regulated gene; CRYAB, αB-crystallin; HSPB2, heat shock 27 kDa protein 2; FBS, fetal bovine serum; GAPDH, glyseraldehyde-3-phosphate dehydrogenase; RACE, rapid amplification of cDNA ends; RT-PCR, reverse transcription-polymerase chain reaction.

## Authors' contributions

PYC designed and performed the experiments, and analyzed the data. RHC helped conceive the project and participated in writing the manuscript. YKL assisted in drafting of the manuscript. CYC contributed in the early stages of the work and identification of the bidirectional promoter. SCL helped perform the Northern blot assays. CWW and WSWC supervised the project and oversaw the experiments. All authors read and approved the final manuscript.

## Figures and Tables

**Table 1:** Primer sequences used to amplify intergenic fragments for subsequent cloning

Construct*	**Primer sequence**	
	**(Forward)**	**(Reverse)**
pGL3-Basic constructs		
1. *SERPINI1 *(1-851)	5'-ATAGATCTACTCCGGCGACGCCGGA-3'	5'-ATAAGCTTGTCCAGACTGCGCCTCT-3'
2. *SERPINI1 *(1-175)	5'-ATAGATCTACTCCGGCGACGCCGGA-3'	5'-ATAAGCTTTCGCCTCAGGATCCCCC-3'
3. *SERPINI1 *(176-851)	5'-CGAGATCTTGGGTGGTGACACTTCT-3'	5'-ATAAGCTTGTCCAGACTGCGCCTCT-3'
4. *SERPINI1 *(474-851)	5'-AGAGATCTGTGTGGGGACTTGCACT-3'	5'-ATAAGCTTGTCCAGACTGCGCCTCT-3'
5. *SERPINI1 *(711-851)	5'-ATAGATCTCAGGGCAGCTGCTTTCC-3'	5'-ATAAGCTTGTCCAGACTGCGCCTCT-3'
6. *PDCD10 *(851-1)	5'-ATAGATCTGTCCAGACTGCGCCTCT-3'	5'-ATAAGCTTACTCCGGCGACGCCGGA-3'
7. *PDCD10 *(710-1)	5'-TCAGATCTGTACCTTTCCGCTCCCT-3'	5'-ATAAGCTTACTCCGGCGACGCCGGA-3'
8. *PDCD10 *(473-1)	5'-ATAGATCTCAAGCCCTCCCTTCACC-3'	5'-ATAAGCTTACTCCGGCGACGCCGGA-3'
9. *PDCD10 *(175-1)	5'-ATAGATCTTCGCCTCAGGATCCCCC-3	5'-ATAAGCTTACTCCGGCGACGCCGGA-3'
10. *PDCD10 *(107-1)	5'-ATAGATCTGCCGGCGAGCGGGACTA-3'	5'-ATAAGCTTACTCCGGCGACGCCGGA-3'
11. *PDCD10 *(175-108)	5'-ATAGATCTTCGCCTCAGGATCCCCC-3'	5'-CCAAGCTTGTTGTCCGCAATTCCTG-3'
12. PDCD10 (851-176)	5'-ATAGATCTGTCCAGACTGCGCCTCT-3'	5'-CGAAGCTTTGGGTGGTGACACTTCT-3'
		
Heterologous promoter constructs		
1. *SERPINI1 *(176-851)/S	5'-CCTGCTAGCTGGGTGGTGACACTTCTAG-3'	5'-AAAGATCTGTCCAGACTGCGCCTCTG-3'
2. *PDCD10 *(851-176)/S	5'-TAAGCTAGCGTCCAGACTGCGCCTCTG-3'	5'-CCTAGATCTTGGGTGGTGACACTTCTAG-3'
3. *PDCD10 *(473-176)/S	5'-TATGCTAGCCAAGCCCTCCCTTCACCTGG-3'	5'-CCTAGATCTTGGGTGGTGACACTTCTAG-3'
4. *PDCD10 *(710-474)/S	5'-GCTGCTAGCGTACCTTTCCGCTCCCTG-3'	5'-GAAGATCTGTGTGGGGACTTGCACTG-3'
5. *PDCD10 *(851-711)/S	5'-TAAGCTAGCGTCCAGACTGCGCCTCTG-3'	5'-TTAGATCTCAGGGCAGCTGCTTTCCG-3'
6. *SERPINI1 *(176-473)/C	5'-CCTGCTAGCTGGGTGGTGACACTTCTAG-3'	5'-CCAGATCTCAAGCCCTCCCTTCACCTGG-3'
7. *SERPINI1 *(474-710)/C	5'-GGAGCTAGCGTGTGGGGACTTGCA-3'	5'-CTAGATCTGTACCTTTCCGCTCCCTG-3'
8. *SERPINI1 *(711-851)/C	5'-CTTGCTAGCCAGGGCAGCTGCTTTCCG-3'	5'-AAAGATCTGTCCAGACTGCGCCTCTG-3'

*Construct names with an "S" character at the end indicates that the PCR-amplified intergenic product was cloned into the upstream of SV40 minimal promoter. Construct names with a "C" character denotes that the amplified intergenic segment was cloned into the upstream of CMV promoter.
